# Quantifying disease activity in rheumatoid arthritis with the TSPO PET ligand ^18^F-GE-180 and comparison with ^18^F-FDG and DCE-MRI

**DOI:** 10.1186/s13550-019-0576-8

**Published:** 2019-12-19

**Authors:** Marius de Groot, Neel Patel, Roido Manavaki, Robert L. Janiczek, Mats Bergstrom, Andrew Östör, Danielle Gerlag, Alexandra Roberts, Martin J. Graves, Yakshitha Karkera, Disala Fernando, Prafull Mistry, Adam Walker, Nicolas Wisniacki, Tim D. Fryer, Pilar Jimenez-Royo

**Affiliations:** 1 Clinical Pharmacology and Experimental Medicine, GlaxoSmithKline R&D, Gunnels Wood Road, Stevenage, UK; 2 Department of Radiology, University of Cambridge, Cambridge, UK; 3 Monash University, Cabrini Medical Centre, Melbourne, Australia; 4 RxCelerate, Cambridge, UK; 5 Biostatistics, GlaxoSmithKline R&D, Bangalore, India; 6 Clinical Unit Cambridge (CUC), GlaxoSmithKline R&D, Addenbrooke’s Hospital, Cambridge, UK; 7 Wolfson Brain Imaging Centre, Department of Clinical Neurosciences, University of Cambridge, Cambridge, UK

**Keywords:** PET, TSPO, FDG, MRI, Rheumatoid arthritis

## Abstract

**Purpose:**

While the aetiology of rheumatoid arthritis (RA) remains unclear, many of the inflammatory components are well characterised. For diagnosis and therapy evaluation, in vivo insight into these processes would be valuable. Various imaging probes have shown value including dynamic contrast-enhanced (DCE) MRI and PET/CT using ^18^F-fluorodeoxyglucose (^18^F-FDG) or tracers targeting the translocator protein (TSPO). To evaluate ^18^F-GE-180, a novel TSPO PET tracer, for detecting and quantifying disease activity in RA, we compared ^18^F-GE-180 uptake with that of ^18^F-FDG and DCE-MRI measures of inflammation.

**Methods:**

Eight RA patients with moderate-to-high, stable disease activity and active disease in at least one wrist were included in this study (NCT02350426). Participants underwent PET/CT examinations with ^18^F-GE-180 and ^18^F-FDG on separate visits, covering the shoulders and from the pelvis to the feet, including hands and wrists. DCE-MRI was performed on one affected hand. Uptake was compared visually between tracers as judged by an experienced radiologist and quantitatively using the maximum standardised uptake value (SUV_max_). Uptake for both tracers was correlated with DCE-MRI parameters of inflammation, including the volume transfer coefficient *K*^trans^ using Pearson correlation (r).

**Results:**

PET/CT imaging with ^18^F-GE-180 in RA patients showed marked extra-synovial uptake around the affected joints. Overall sensitivity for detecting clinically affected joints was low (14%). ^18^F-GE-180 uptake did not or only weakly correlate with DCE-MRI parameters in the wrist (*r* = 0.09–0.31). ^18^F-FDG showed higher sensitivity for detecting symptomatic joints (34%), as well as strong positive correlation with DCE-MRI parameters (SUV_max_ vs. *K*^trans^: *r* = 0.92 for wrist; *r* = 0.68 for metacarpophalangeal joints).

**Conclusions:**

The correlations between DCE-MRI parameters and ^18^F-FDG uptake support use of this PET tracer for quantification of inflammatory burden in RA. The TSPO tracer ^18^F-GE-180, however, has shown limited use for the investigation of RA due to its poor sensitivity and ability to quantify disease activity in RA.

## Introduction

Rheumatoid arthritis (RA) is a systemic autoimmune, erosive arthropathy affecting 0.5–1% of the general population [1]. If untreated, the condition can progressively lead to severe damage of articular and periarticular structures, significant disability, multiple comorbidities and an overall reduction in life expectancy [2, 3]. Although the aetiology of RA remains unclear, the complex interaction of several proinflammatory cells, including macrophages and mediators, is the basis of disease pathogenesis. The net effect of these interactions is synovial proliferation, sustained inflammation, increased microvascular permeability and hyperaemia, erosion of articular cartilage and bone, tissue matrix degradation and eventual articular destruction [4, 5].

To quantify the inflammatory burden in RA non-invasively, imaging using a variety of modalities including magnetic resonance imaging (MRI) and positron emission tomography computed tomography (PET/CT) has been used. PET/CT with ^18^F-fluorodeoxyglucose (^18^F-FDG) has been used to assess inflammation, as an increase in ^18^F-FDG uptake is associated with immune cell activation and recruitment. ^18^F-FDG uptake has been shown to correlate with biochemical and clinical markers of RA severity [6–14] and clinical outcome [15] and has been used for the assessment of response to therapy [15–22]. Nevertheless, as a tracer of glucose metabolism, ^18^F-FDG is a non-specific marker of inflammation and is, e.g. in arthropathies also taken up by non-inflammatory cells [9]. Similarly, dynamic contrast-enhanced MRI (DCE-MRI) has been used to study RA joint tissues and provides an established measure of local severity of disease [23, 24]. Simpler contrast-enhanced MRI endpoints [25, 26] are in fact used routinely for assessing synovitis in intervention studies [23, 27, 28]. While more involved to implement, DCE-MRI is the more sensitive of the methods and able to assess synovial perfusion/vascular permeability, vascular volume and interstitial volume; all of which can be affected by chronic inflammation. While DCE-MRI can provide meaningful insight in the local inflammatory status in RA, it can only measure a small field of view, e.g. a single knee or wrist, and, like ^18^F-FDG, its signal is not specific for any cell type involved in RA. Both ^18^F-FDG PET and DCE-MRI have been used previously to study joint synovial inflammation in RA, but the two techniques have not been compared in a single study.

Macrophages have a key role in RA, and in vivo quantification would provide a valuable marker for tracking disease activity and response to therapy. This has led to the investigation of PET imaging with tracers targeting translocator protein (TSPO), an outer-membrane mitochondrial protein ubiquitously expressed on activated macrophages [29–32]. ^18^F-GE-180 [33] (flutriciclamide™; GE Healthcare) is a novel TSPO ligand that has shown improved specificity for TSPO compared to ^11^C-PK11195 in preclinical studies [34, 35]. This tracer has been previously evaluated for diagnosis and monitoring of inflammation in neurological and neurodegenerative pathologies, including multiple sclerosis, stroke and Alzheimer’s disease [34–36]. It has, however, not yet been tested in rheumatic diseases such as RA. The aim of this pilot study was therefore to identify and characterise inflammation in RA patients based on TSPO and abnormal glucose metabolism with ^18^F-GE-180 and ^18^F-FDG PET/CT, using DCE-MRI to correlate perfusion-based measures of inflammation with tracer uptake for both tracers. This study used an adaptive design with an interim futility analysis to allow poor initial observations with ^18^F-GE-180 imaging to trigger early termination.

## Materials and methods

### Patient population

Nine patients were recruited by advertisement and from the outpatient population of Addenbrooke’s Hospital, Cambridge, UK. For inclusion, patients had to fulfil either the 1987 American College of Rheumatology definition or the 2010 American College of Rheumatology/European League Against Rheumatism (ACR/EULAR) classification criteria for RA [37]. These RA patients needed to have stable disease with moderate-to-high activity as defined by a disease activity score of 28 joints [38], using the erythrocyte sedimentation rate (DAS28-ESR), of ≥ 3.2 at screening and at least one painful or swollen wrist joint as assessed by a rheumatologist. Patients receiving treatment with biologics were excluded from the study. One patient was withdrawn at the discretion of the investigator (DF) following the incidental finding of a lung lesion that required separate follow-up which precluded continuation in the study, leaving eight patients for analysis.

The study (trial NCT02350426, GSK study 201659) was approved by a National Research Ethics Committee (REC no 14/SC/1405) and the Administration of Radioactive Substances Advisory Committee (ARSAC), UK. All subjects gave written informed consent before participation in the study.

### Clinical evaluation

Joint swelling and tenderness were assessed for all joints included in the DAS28 score. Laboratory markers of inflammation, including ESR and C-reactive protein (CRP), were also measured. Joint scores, ESR and a patient global health assessment were used to calculate the DAS28-ESR score, which was assessed at screening to determine inclusion and again at both imaging visits. All clinical evaluations were performed by a rheumatologist at screening and by a specialist nurse trained in joint assessment at the study visits. In addition, the joint assessments permitted a binary classification into symptomatic (swollen and/or tender) and asymptomatic joints. Given the effect of the Ala147Thr polymorphism on TSPO-binding affinity for ^18^F-GE-180, all participants were tested for presence of this polymorphism, and individuals with predicted low-binding affinity were excluded from the study [39]. Medical history, demographics and concomitant medication information were also collected.

### Imaging protocol

*PET/CT*: Examinations were undertaken on a GE Discovery 690 PET/CT (GE Healthcare; Waukesha, WI), Addenbrooke’s Hospital, UK. Participants underwent two PET/CT scans, one with ^18^F-FDG (Alliance Medical, UK) and the other with ^18^F-GE-180 (GE Healthcare, Amersham, UK), ordered randomly, with the second scan occurring 1–15 days after the first scan (median: 8 [range: 6–15] days). PET scans involved intravenous administration of 159 ± 7 MBq ^18^F-FDG or 189 ± 6 MBq ^18^F-GE-180. Administration of both tracers was followed by an uptake period of 60 min (median: 60.2 [range: 59.4–70.2] min). A low-dose CT (120 kVp 30 mA, 1.375 pitch) was acquired immediately prior to each PET acquisition for attenuation correction and anatomical localisation. PET imaging of the lower body, extending from the pelvis to the bottom of the feet, included 8–10 axial bed positions; 5 min/bed position. Subjects were imaged in the supine position with arms down to ensure coverage of the hand and wrist area, with hands lightly bandaged to acrylic positioning devices to allow for fair comparison between subjects and imaging modalities. A separate 5-min single-bed acquisition of the shoulder area was performed starting 120 min (median: 119.6 [range: 119.1–120.6] min) post tracer administration. Prior to ^18^F-FDG injection, patients were required to fast for at least 6 hr. Fasted blood glucose concentration was tested in all participants to ensure levels were below 7 mmol/L.

Emission data were reconstructed into a 256 × 256 × 47 matrix with 2.7 × 2.7 × 3.27 mm voxels, using time-of-flight ordered subsets-expectation maximisation (TOF-OSEM) with 4 iterations and 24 subsets. Corrections were applied for attenuation, scatter, randoms, dead time, sensitivity, normalisation and isotope decay, together with a 6-mm full width at half maximum Gaussian filter post reconstruction.

*MRI*: Imaging of the right wrist (which was deemed the most affected in all subjects’ clinical assessments) was performed on a 3 T MR system (MR750, GE Healthcare; Waukesha, WI), Addenbrooke’s Hospital, Cambridge, UK, using an 8-channel knee coil. Patients were scanned in the prone position, with the arm of the affected hand extending upwards above their head. The hand was positioned in the centre of the coil, with the coil in the centre of the magnet to ensure good signal in both the wrist and metacarpophalangeal joints. The hand was lightly bandaged to the same positioning device used during PET/CT scanning and placed inside the coil to minimise motion during scanning.

The imaging protocol included a coronal pre-contrast fat-suppressed (FS) *T*_*1*_-weighted 3D spoiled gradient echo (3D SPGR) acquisition for radiological assessment as well as for shape modelling and ROI definition. This sequence was repeated post-contrast at the end of the imaging session. For DCE-MRI, 3D variable flip angle SPGR acquisitions were acquired first to allow baseline *T*_*1*_-mapping, followed by a 7-minute dynamic 3D SPGR sequence, during which gadolinium (Gd) contrast agent was administered. A dose of 0.1 mmol/kg body weight of gadobutrol (Gadovist, Bayer Schering Pharma, Berlin, Germany) was injected using a power injector, followed by a 25 mL flush of saline. Both volumes were injected at 3.0 mL/s, with injection of the contrast agent at the sixth measurement of the dynamic series. Pulse sequence parameters are described in Table 1. Total scan time was limited to < 30 min to keep the potential for patient discomfort to a minimum.

**Table 1:** MRI scanning parameters for structural and DCE-MR imaging

	3D SPGR + FS PRE/POST-Gd	3D DCE-MRI
Scan plane	Coronal	Coronal
TE (ms)	8.3	1.2
TR (ms)	30	3.5
Flip angles (deg)	30	6, 2, 14 for *T*_*1*_-mapping, 14 for dynamic series
FOV (cm)	18	18
Phase FOV	0.75	0.75
Slice thickness (mm)	1.5	1.5
Slices acquired	40	40
Measurements	-	8 for *T*_*1*_-mapping, 54 for dynamic series
Acquisition matrix	512 × 288	180 × 136
Saturation	Fat suppression	-

### Image quantification and scoring

For both PET tracers, both qualitative and quantitative evaluations were performed by an experienced radiologist (NP). For qualitative evaluation, joints were inspected visually with uptake compared to uptake in adjacent subcutaneous tissue and bone (marrow). Uptake was categorised using a three-point scale: normal, possibly abnormal and definitely abnormal. Quantitative analysis involved calculation of maximum standardised uptake values (SUV_max_), normalised to body weight, identified as the maximum inside a sphere encompassing the joint, while making sure that the location of the maximum was inside the synovium. Joints incorporated in the DAS28 calculation, including the shoulders, wrists, metacarpophalangeal and proximal interphalangeal joints and knees, as well as the distal interphalangeal joints the hips and ankles, were evaluated. The elbows were not imaged as part of this study, giving 38 joints evaluated per patient qualitatively and quantitatively. The hips of one patient could not be evaluated, and one hip of a single subject was excluded from evaluation as it had been replaced by a joint prosthesis. All assessments were performed with the reviewer blinded to clinical and other imaging data.

For the DCE-MRI analysis, all images were centrally checked for image quality with any images suffering from operator error or image artefacts discarded. Regions of interest of wrist and metacarpophalangeal joint synovial spaces were defined on the pre-contrast 3D SPGR+FS images using active appearance models [40] which were previously built from an independent training set of subjects (RA patients and healthy volunteers). Pharmacokinetic analysis of DCE-MRI data utilised the standard Tofts model [41] for the calculation of the volume transfer coefficient, *K*^trans^, as previously described [23]. Also, a simpler data-driven model was fitted to the DCE-MRI time series to yield estimates for the initial rate of enhancement (IRE) and maximal enhancement (ME). The volume of the enhancing pannus (VEP) was calculated from the difference between the pre- and post-contrast 3D SPGR+FS images to identify regions of enhancement in the joint and normalised to the total synovial volume (V_total_) as VEP/V_total_. Scanner specific *T*_*1*_ correction of the DCE-MRI data was also performed using phantom data, as described previously [23].

3D DCE-MRI parametric maps were evaluated in an ROI based on the VEP for each joint of interest, with non-enhancing voxels excluded from both models. Voxelwise parameter estimates were averaged for *K*^trans^, IRE and ME for each joint.

### Statistical analysis

This was an exploratory study and therefore there were no formal sample size calculations. For qualitative analysis, clinical symptoms and tracer uptake were cross-tabulated. Treating symptoms as positive/negative (P/N) and tracer increase as a detection test with true/false (T/F) labels, commonly defined performance measures were computed as follows: sensitivity = TP / (TP + FN), specificity = TN / (TN + FP), positive predictive value = TP / (TP + FP) and negative predictive value = TN / (TN + FN). Lastly, the agreement between visual scores of both tracers was compiled in a confusion matrix.

To evaluate ^18^F-FDG and ^18^F-GE-180 for the assessment of disease severity, quantitative measures of tracer uptake in the wrist and metacarpophalangeal joints were correlated with DCE-MRI measures using Pearson correlation (r). Primarily, our analysis focused on correlations with *K*^trans^, as a marker of local severity of disease, but also included other DCE-MRI parameters (VEP; VEP/V_total_; IRE; ME).

All analyses were performed as available-case analysis to deal with sporadic missing values caused by failure of the DCE-MRI model to converge or clinical assessments that could not be performed. Analyses were performed using SAS software (Version 9.4 m3, SAS Institute Inc., Cary, NC, USA). Data are presented as number (%), mean ± standard deviation (SD) or median [range] as appropriate.

## Results

### Demographics

The mean age of the subjects was 54 ± 12.1 years. Three of the patients were female (38%). The mean body mass index (BMI) of the subjects was 28.4 ± 4.6 kg/m^2^. Further demographic information is given in Table 2, which is generated post hoc to only summarise the imaging population.

**Table 2:** Participant demographics

	Overall (*N* = 8)
Age (years)	54 (12.1)
Sex	
Female	3 (38)
Male	5 (63)
BMI (kg/m^2^)	28.4 (4.6)
Height (m)	1.75 (0.08)
Weight (kg)	85.7 (9.4)
Race	
White-White/Caucasian/European Heritage	8 (100)
DAS28-ESR	5.4 (0.9)
Tender joints median [range]	14.5 [8–27]
Swollen joints median [range]	3.5 [2–9]
ESR (mm)	17.5 (11.1)
CRP (mg/L)	7.3 (8.5)
Median [range]	3.0 [1.5–24.5]
Binding affinity for TSPO	
Mixed affinity	4 (50)
High affinity	4 (50)

Values expressed as mean (SD) or n (%) as appropriate unless indicated differently. Values determined at screening. BMI indicates body mass index, DAS disease activity score, ESR erythrocyte sedimentation rate, CRP C-reactive protein, TSPO translocator protein, SD standard deviation

### Qualitative analysis

**Fig. 1:**
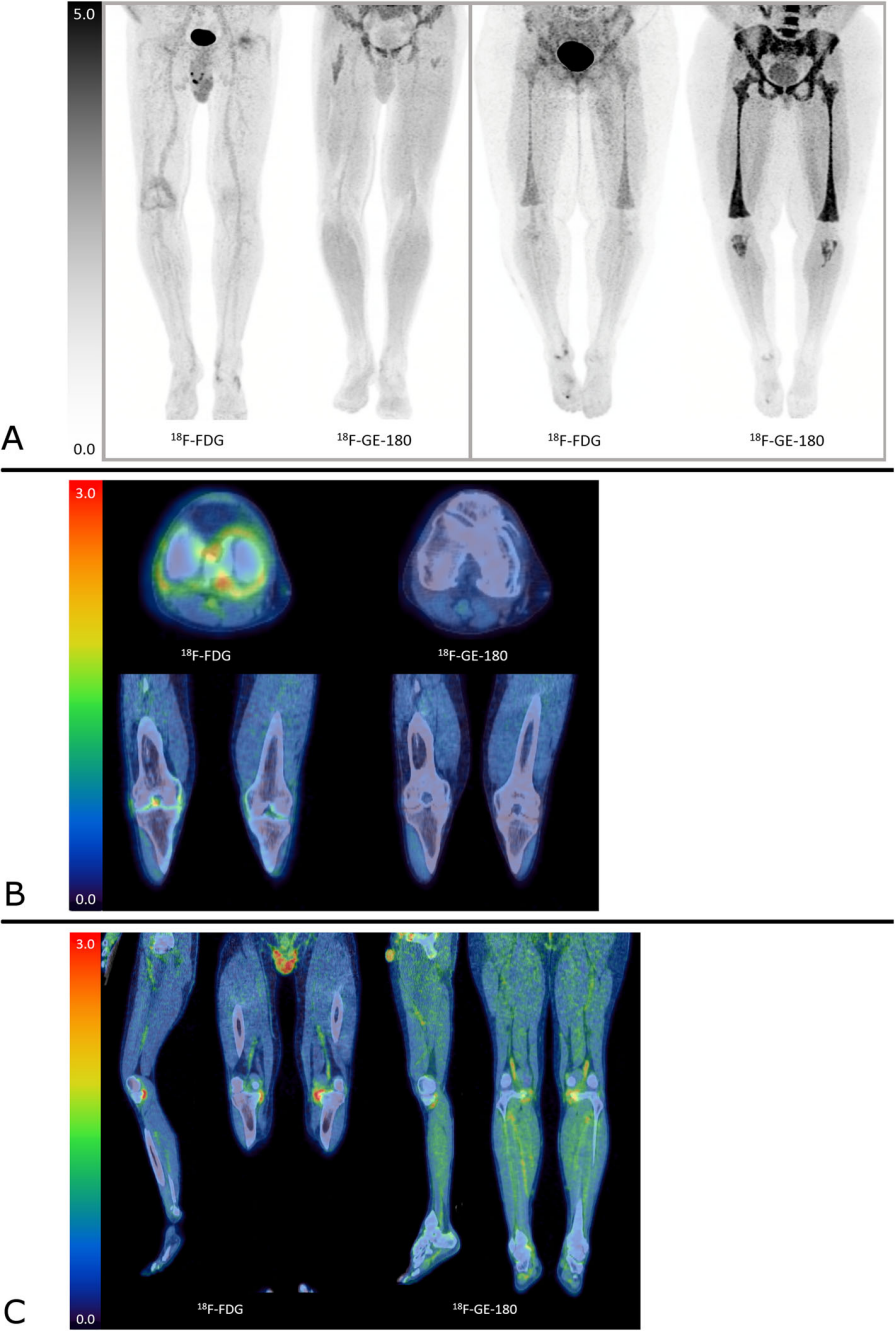
Representative imaging. **a** demonstrates maximum intensity projections (MIPs) of two patients imaged with ^18^F-FDG and ^18^F-GE-180. The two left images are of a patient who has increased right knee joint uptake with ^18^F-FDG, not seen with ^18^F-GE-180, which is also demonstrated on axial and coronal fused images in **b**. The images with ^18^F-GE-180 demonstrate a typical pattern of physiological uptake with marrow uptake in the proximal femurs and diffuse muscle uptake. The two right images of Fig. **a** are of a separate patient who demonstrates marked uptake within the marrow down to the proximal tibiae with ^18^F-GE-180. A similar but less intense uptake is seen with ^18^F-FDG and is presumed related to a reactive marrow. **c** are coronal and sagittal images of a patient with increased uptake in the medial aspects of both knee joints which is much more marked with ^18^F-FDG, compared to ^18^F-GE-180. The ^18^F-GE-180 images also demonstrate increased muscle and marrow uptake. The scale on all figures is SUV (g/mL)

Observations on the ^18^F-GE-180 images include substantial but variable activity in muscle and vasculature, which was more pronounced than for ^18^F-FDG. There was also prominent activity within the marrow. In particular, in one patient there was marked activity in the marrow extending from the spine to the knees and proximal tibiae (Fig. 1). This uptake corresponded with similar but less avid uptake on the ^18^F-FDG scan and was suggestive of a reactive marrow. In general, ^18^F-GE-180 uptake in clearly affected joints was much less than expected and often only subtly raised against background. Example images are displayed in Fig. 1 a–c. The variability in appearance made it difficult to suggest an appropriate reference tissue for this tracer.

A definite increase in the visual score was observed for ^18^F-FDG uptake in 46 (15%) joints as compared with 11 (4%) joints for ^18^F-GE-180. Possible increase was identified in 28 (9%) and 22 (7%) joints for ^18^F-FDG and ^18^F-GE-180, respectively. When constrained to joints included in the clinical assessment of the DAS28 score, most of the symptomatic joints did not show increased scores for either tracer on visual inspection, even when grouping ‘definite’ and ‘possible’ increase categories. Summarised results for these joints are shown in Table 3 (post hoc analysis). Performance measures including specificity and positive and negative predictive values of visual scores for both tracers are listed in Table 4 (post hoc analysis). Sensitivity for detecting symptomatic joints was 34% for ^18^F-FDG and 14% for ^18^F-GE-180.

**Table 3:** Cross-tabulation of PET-CT visual score and clinical status

	^18^F-FDG visual score			^18^F-GE-180 visual score		
	Normal	Increased	*Total*	Normal	Increased	*Total*
Symptomatic joints	74	38	*112*	100	16	*116*
Asymptomatic joints	70	26	*96*	80	12	*92*
*Total*	*144*	*64*	*208*	*180*	*28*	*208*

Joints that were swollen and/or tender were regarded symptomatic. Joints in DAS28 examination were included; elbows were not imaged

**Table 4:** PET-CT visual score and clinical status, performance at identifying symptomatic joints

	^18^F-FDG			^18^F-GE-180		
Sensitivity (%)	34			14		
Specificity (%)	73			87		
Positive predictive value (%)	59			57		
Negative predictive value (%)	49			44		

There was a fair correspondence between visual scores for both tracers, shown in Table 5. While most joints were classified as ‘normal’ for both tracers, the majority of joints that showed increased ^18^F-GE-180 uptake also showed increased ^18^F-FDG uptake. Conversely however, only a minority of joints that showed increased ^18^F-FDG uptake also showed increased ^18^F-GE-180 uptake.

**Table 5:** Agreement between visual uptake scores of both tracers

		^18^F-GE-180		
		normal	increased	total
^18^F-FDG	normal	223	6	229
	increased	47	27	74
	total	270	33	303

Joints included in the DAS28, plus the distal interphalangeal joints, the hips, knees and ankles were evaluated. The elbows were not imaged. See Appendix for note on corrected number of joints included

### Quantitative analysis

**Fig. 2:**
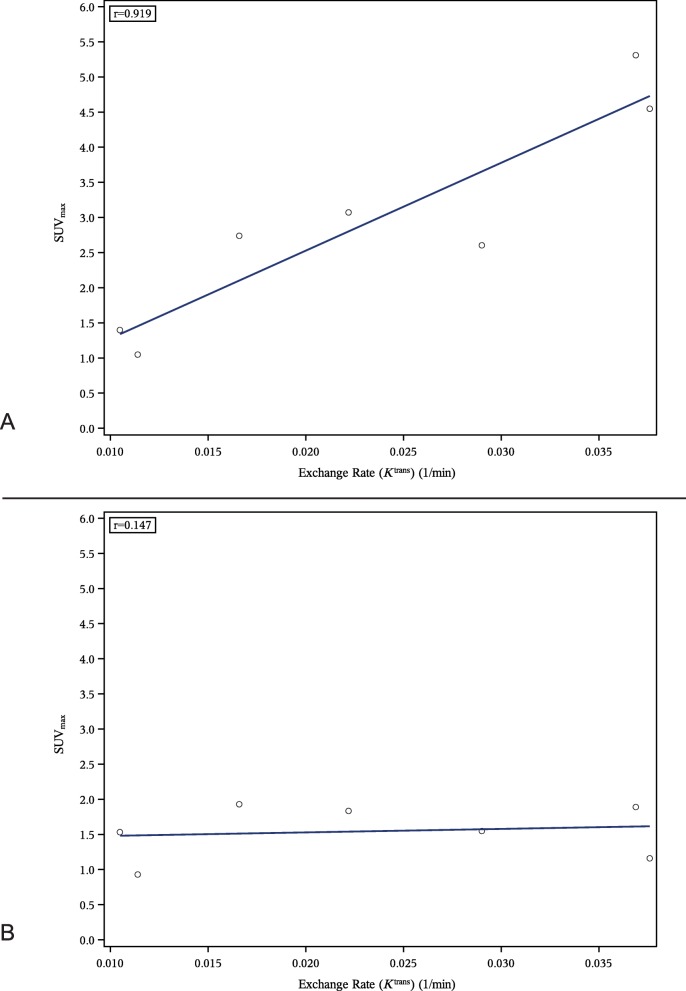
Correlation between K^trans^ and SUV_max_ in wrist joints for ^18^F-FDG (**a**) and ^18^F-GE-180 (**b**)

**Fig. 3:**
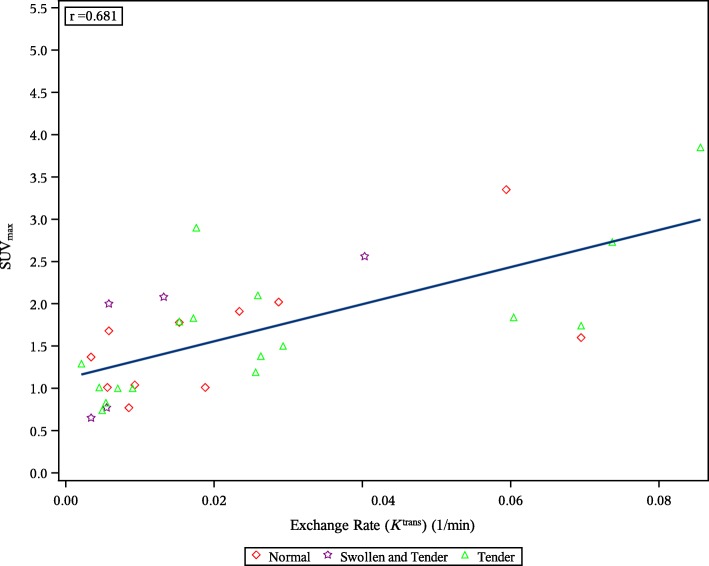
Correlation between K^trans^ and SUV_max_ in metacarpophalangeal joints for ^18^F-FDG

Correlations between PET and DCE-MRI measures in the hand showed a strong correlation between ^18^F-FDG SUV_max_ in the wrist and DCE-MRI *K*^trans^ as shown in Fig. 2a (post hoc analysis) and reflected in a Pearson correlation coefficient (r) of 0.92. The same analysis for ^18^F-GE-180 showed no correlation (*r* = 0.15), Fig. 2b (post hoc analysis). Correlations for other DCE-MRI parameters were comparable for both tracers and are listed in the first columns of Table 6 (post hoc analysis). Correlations for metacarpophalangeal joints showed slightly weaker correlations for ^18^F-FDG (*r* = 0.68 for *K*^trans^) than for the wrist, and weak correlations for ^18^F-GE-180 (*r* = 0.38 for *K*^trans^), shown in the last columns of Table 6 (post hoc analysis). Figure 3 (post hoc analysis) shows an example correlation plot for the metacarpophalangeal joints for correlation between *K*^trans^ and ^18^F-FDG SUV_max_ as a representative illustration of the distribution of the measurements.

**Table 6:** Pearson correlation coefficients for correlations between DCE-MRI parameters and SUV_max_ for ^18^F-FDG (A) and ^18^F-GE-180 (B)

	Wrist joint	MCP joints
A ^18^F-FDG		
*K*^trans^	0.92	0.68
VEP/Vtotal	0.90	0.82
IRE	0.92	0.71
ME	0.92	0.70
B ^18^F-GE-180		
*K*^tran*s*^	0.15	0.38
VEP/Vtotal	0.31	0.47
IRE	0.13	0.36
ME	0.09	0.29

MCP indicates metacarpophalangeal

## Discussion

In this exploratory pilot study on combined molecular imaging techniques and DCE-MRI for RA, we found strong positive correlations (range: *r* = 0.7–0.9) between severity of disease as measured by DCE-MRI parameters and glucose metabolism measured with ^18^F-FDG across hand and wrist joints. For the novel TSPO ligand ^18^F-GE-180, these correlations were weak (range: *r* = 0.1–0.5). When visually identifying clinically symptomatic joints, a comparison between ^18^F-FDG and ^18^F-GE-180 suggested a marginally higher specificity in detecting RA pathology for the TSPO ligand (73% and 87%, respectively) but with a lower sensitivity (34% and 14%, respectively). The poor ability to identify symptomatic joints or disease activity in symptomatic joints suggests a poor applicability of ^18^F-GE-180 for disease quantification in RA.

Considering the systemic nature of RA and that macrophage synovial infiltration can be present without clinically active rheumatoid arthritis [42, 43], it was hypothesised that ^18^F-GE-180 would demonstrate better sensitivity than ^18^F-FDG in identifying joints with subclinical synovial tissue inflammation. Through its mechanistic involvement by binding to the macrophages that are pivotal in the pathogenesis of RA, it was also hypothesised that ^18^F-GE-180 would offer increased specificity to inflammation against, e.g. osteoarthritic degeneration.

Earlier reports on the use of TSPO PET tracers in RA indeed suggest applicability in imaging of the inflamed synovium in RA. Van der Laken et al. [29] previously showed higher expression of TSPO with ^11^C-PK11195 in RA patients with severe synovial swelling of the knee compared to patients with mild swelling of the knee, correlating with TSPO and the macrophage marker CD68 on immunohistochemistry. In addition, tracer uptake in contralateral uninflamed knee joints of these patients was significantly higher than in uninflamed joints of control patients without inflammatory joint disease, suggesting the ability to demonstrate presence of subclinical disease activity. More specifically, Narayan et al. [32] used autoradiography and PET imaging with PBR-28 ligands in patients with RA and healthy controls and found elevated TSPO expression by fibroblast-like synoviocytes and activated macrophages in the synovium of RA patients. In our study, we explored the utility of ^18^F-GE-180, a TSPO tracer with higher selectivity for TSPO than ^11^C-PK11195 [33], for the quantification of joint inflammation in RA as a potential tool for evaluating response to (novel) therapies in RA. This is the first study testing this novel tracer for a non-neurological indication, and the relevant scanning parameters were selected on the basis of preclinical data [35] and discussions with GE Healthcare where initial human data were shown. The chosen time window for the study is consistent with published work in the brain [44–46], which indicates time-activity curves reaching a plateau after ~ 40–45 min post-injection and good agreement between SUV ratio and Logan distribution volume ratio for time points later than 60 min.

This exploratory study used an adaptive design, offering an interim futility analysis. While the original target for this study was the inclusion of 20 patients, the unsatisfactory performance of ^18^F-GE-180, as shown in the imaging results presented here, led to early termination of the study.

The strengths of this study are the side-by-side comparison of ^18^F-FDG and DCE-MRI to assess synovitis in RA for the first time, the use of a well-characterised patient cohort and the evaluation of a novel TSPO tracer to image macrophage burden. The limitations are the reliance on imaging measures to determine synovial inflammation without having the capability to confirm this by tissue analysis, the absence of a control-group without RA and the limited sample size. A consequence of the early termination of this study was that only two participants underwent the optional dynamic PET imaging using ^18^F-GE-180, with both data sets non-analysable for technical reasons. Unavailability of dynamic imaging data thus prevented us to investigate pharmacokinetics and to verify static imaging parameters.

In RA, clinically non-inflamed joints can show signs of inflammation when analysing the synovial tissue [43]. Although not confirmed by tissue analysis, in our study 27% and 13% of asymptomatic, clinically non-inflamed joints showed visible signs of inflammation on^18^F-FDG and ^18^F-GE-180 imaging, respectively. The poor sensitivity measures observed in this study highlight the failure of both tracers to detect a substantial portion of clinically symptomatic joints (Table 4).

The reported sensitivity of ^18^F-FDG for detecting inflammatory arthritis, as based on clinical assessment, varies between 56% and 77% [47]. In a study of 21 patients with active RA, positivity using ^18^F-FDG was found in 63% of joints, whereas 75% and 79% were positive for swelling and tenderness analysis, respectively [6]. The ^18^F-FDG sensitivity found in our study of 34% was lower. While the mean DAS28 score of 5.4 is representative for an RA patient population with moderate-to-high disease activity, the median CRP value found in our study was relatively low (3.0 (range: 1.5–24.5) mg/L) when compared to values found in the literature [6, 7, 48, 49]. It is possible that the low CRP levels in our study reflect a population with milder systemic inflammatory activity, which could in part explain the overall lower level of ^18^F-FDG sensitivity.

The difference in the number of symptomatic joints and joints showing synovial uptake of ^18^F-FDG may be explained by the difference in definitions of ‘abnormal ^18^F-FDG uptake’. In this study, abnormality was defined by comparison with adjacent structures, whereas studies reported in the literature used comparisons with reference tissues, such as the liver and brain [13, 48]. In our study, some joints were not classified as abnormal despite having uptake greater than the liver because uptake was considered spurious due to, e.g. spillover of uptake from adjacent structures (e.g., bone marrow) and increased noise from joints being at the edge of the field of view. In addition, abnormal uptake in our study was defined as synovial uptake, while uptake in associated structures such as musculotendinous junctions was disregarded. It is possible that uptake in these adjacent structures of a joint could represent features that clinically may be assessed as a symptomatic (swollen and/or tender) joint.

In our study, ^18^F-FDG PET quantified inflammatory disease activity well when using DCE-MRI as comparison. This observation strengthens confidence in both imaging probes but, in combination with the poor correlation found between markers of systemic inflammation and clinical symptomatology, also highlights the distinct aspects of disease probed clinically and through imaging. The results of our study suggest that ^18^F-FDG PET as well as DCE-MRI can add robust information on disease activity to the clinical evaluation of joints.

The data from our study are in broad agreement with some published preclinical studies [50], but even though previous reports in RA with ^11^C-PK11195 and more recently PBR-28 ligands have shown more encouraging results [29, 32], our results do not support use of ^18^F-GE-180 in the RA population investigated. Without biopsy data, it is not possible to know whether the low uptake of ^18^F-GE-180 was the result of low TSPO expression in the synovial tissue perhaps due to a less inflammatory population or poor performance by the tracer.

## Conclusion

In this study, ^18^F-FDG PET-CT visual scores showed limited ability in identifying symptomatic joints in RA, but quantitative interpretation revealed a high agreement with DCE-MRI measures in quantifying disease activity in the hands. While ^18^F-GE-180 PET-CT might be slightly more specific at detecting symptomatic joints in RA, its poor sensitivity and ability to quantify synovial inflammation in RA will mostly preclude further use of the tracer in this disease.

## Data Availability

Anonymised individual participant data and study documents can be requested for further research from www.clinicalstudydatarequest.com.

## Appendix

Due to an acquisition problem, the hips of one patient could not be evaluated, but they were erroneously scored as ‘normal’ on ^18^F-GE-180. As a result, the normal-normal cell in Table 5 should therefore read 221, instead of 223. Percentages for joints with uptake for both tracers mentioned in the text are unchanged.

## References

[1] Gabriel SE (2001). The epidemiology of rheumatoid arthritis. Rheum Dis Clin North Am..

[2] Pincus T, Callahan LF, Sale WG, Brooks AL, Payne LE, Vaughn WK (1984). Severe functional declines, work disability, and increased mortality in seventy-five rheumatoid arthritis patients studied over nine years. Arthritis Rheum..

[3] Gabriel SE, Michaud K (2009). Epidemiological studies in incidence, prevalence, mortality, and comorbidity of the rheumatic diseases. Arthritis Res Ther..

[4] Choy EHS, Panayi GS (2001). Cytokine pathways and joint inflammation in rheumatoid arthritis. Epstein FH, editor. N Engl J Med..

[5] Scott DL, Wolfe F, Huizinga TW (2010). Rheumatoid arthritis. Lancet..

[6] Beckers C, Ribbens C, André B, Marcelis S, Kaye O, Mathy L (2004). Assessment of disease activity in rheumatoid arthritis with (18)F-FDG PET. J Nucl Med..

[7] Beckers C, Jeukens X, Ribbens C, André B, Marcelis S, Leclercq P (2006). 18F-FDG PET imaging of rheumatoid knee synovitis correlates with dynamic magnetic resonance and sonographic assessments as well as with the serum level of metalloproteinase-3. Eur J Nucl Med Mol Imaging..

[8] Vogel WV, van Riel PLCM, Oyen WJG (2007). FDG-PET/CT can visualise the extent of inflammation in rheumatoid arthritis of the tarsus. Eur J Nucl Med Mol Imaging..

[9] Elzinga EH, van der Laken CJ, Comans EFI, Lammertsma AA, Dijkmans BAC, Voskuyl AE (2007). 2-Deoxy-2-[F-18]fluoro-D-glucose joint uptake on positron emission tomography images: rheumatoid arthritis versus osteoarthritis. Mol Imaging Biol..

[10] Fonseca A, Wagner J, Yamaga LI, Osawa A, Da Cunha ML, Scheinberg M (2008). (18) F-FDG PET imaging of rheumatoid articular and extraarticular synovitis. J Clin Rheumatol.

[11] Ju JH, Kang KY, Kim IJ, Yoon JU, Kim H-S, Park S-H (2008). Visualization and localization of rheumatoid knee synovitis with FDG-PET/CT images. Clin Rheumatol..

[12] Kubota K, Ito K, Morooka M, Mitsumoto T, Kurihara K, Yamashita H (2009). Whole-body FDG-PET/CT on rheumatoid arthritis of large joints. Ann Nucl Med..

[13] Kubota K, Ito K, Morooka M, Minamimoto R, Miyata Y, Yamashita H (2011). FDG PET for rheumatoid arthritis: basic considerations and whole-body PET/CT. Ann N Y Acad Sci..

[14] Basu S, Shejul Y (2014). Regional Lymph node hypermetabolism corresponding to the involved joints on FDG-PET in newly diagnosed patients of rheumatoid arthritis: observation and illustration in symmetrical and asymmetric joint involvement. Rheumatol Int..

[15] Elzinga EH, van der Laken CJ, Comans EFI, Boellaard R, Hoekstra OS, Dijkmans BAC (2011). 18F-FDG PET as a tool to predict the clinical outcome of infliximab treatment of rheumatoid arthritis: an explorative study. J Nucl Med..

[16] Palmer WE, Rosenthal DI, Schoenberg OI, Fischman AJ, Simon LS, Rubin RH (1995). Quantification of inflammation in the wrist with gadolinium-enhanced MR imaging and PET with 2-[F-18]-fluoro-2-deoxy-D-glucose. Radiology..

[17] Polisson RP, Schoenberg OI, Fischman A, Rubin R, Simon LS, Rosenthal D (1995). Use of magnetic resonance imaging and positron emission tomography in the assessment of synovial volume and glucose metabolism in patients with rheumatoid arthritis. Arthritis Rheum..

[18] Chaudhari AJ, Bowen SL, Burkett GW, Packard NJ, Godinez F, Joshi AA (2010). High-resolution 18F-FDG PET with MRI for monitoring response to treatment in rheumatoid arthritis. Eur J Nucl Med Mol Imaging..

[19] Okamura K, Yonemoto Y, Arisaka Y, Takeuchi K, Kobayashi T, Oriuchi N (2012). The assessment of biologic treatment in patients with rheumatoid arthritis using FDG-PET/CT. Rheumatology..

[20] Vijayant V, Sarma M, Aurangabadkar H, Bichile L, Basu S (2012). Potential of 18 F-FDG-PET as a valuable adjunct to clinical and response assessment in rheumatoid arthritis and seronegative spondyloarthropathies. World J Radiol..

[21] Roivainen A, Hautaniemi S, Möttönen T, Nuutila P, Oikonen V, Parkkola R (2013). Correlation of 18F-FDG PET/CT assessments with disease activity and markers of inflammation in patients with early rheumatoid arthritis following the initiation of combination therapy with triple oral antirheumatic drugs. Eur J Nucl Med Mol Imaging..

[22] Okamura K, Yonemoto Y, Okura C, Higuchi T, Tsushima Y, Takagishi K (2014). Evaluation of tocilizumab therapy in patients with rheumatoid arthritis based on FDG-PET/CT. BMC Musculoskelet Disord..

[23] Waterton JC, Ho M, Nordenmark LH, Jenkins M, DiCarlo J, Guillard G (2017). Repeatability and response to therapy of dynamic contrast-enhanced magnetic resonance imaging biomarkers in rheumatoid arthritis in a large multicentre trial setting. Eur Radiol..

[24] Hodgson RJ, Connolly S, Barnes T, Eyes B, Campbell RSD, Moots R (2007). Pharmacokinetic modeling of dynamic contrast-enhanced MRI of the hand and wrist in rheumatoid arthritis and the response to anti-tumor necrosis factor-α therapy. Magn Reson Med..

[25] Østergaard M, Peterfy C, Conaghan P, McQueen F, Bird P, Ejbjerg B (2003). OMERACT rheumatoid arthritis magnetic resonance imaging studies. core set of MRI acquisitions, joint pathology definitions, and the OMERACT RA-MRI scoring system. J Rheumatol..

[26] Østergaard M, Edmonds J, McQueen F, Peterfy C, Lassere M, Ejbjerg B (2005). An introduction to the EULAR–OMERACT rheumatoid arthritis MRI reference image atlas. Ann Rheum Dis..

[27] Peterfy C, Emery P, Tak PP, Østergaard M, DiCarlo J, Otsa K (2016). MRI assessment of suppression of structural damage in patients with rheumatoid arthritis receiving rituximab: results from the randomised, placebo-controlled, double-blind RA-SCORE study. Ann Rheum Dis..

[28] Conaghan PG, Østergaard M, Bowes MA, Wu C, Fuerst T, van der Heijde D (2016). Comparing the effects of tofacitinib, methotrexate and the combination, on bone marrow oedema, synovitis and bone erosion in methotrexate-naive, early active rheumatoid arthritis: results of an exploratory randomised MRI study incorporating semiquantitative and quantitative techniques. Ann Rheum Dis..

[29] van der Laken CJ, Elzinga EH, Kropholler MA, Molthoff CFM, van der Heijden JW, Maruyama K (2008). Noninvasive imaging of macrophages in rheumatoid synovitis using ^11^ C-( *R* )-PK11195 and positron emission tomography. Arthritis Rheum..

[30] Gent YYJ, Voskuyl AE, Kloet RW, van Schaardenburg D, Hoekstra OS, Dijkmans BAC (2012). Macrophage positron emission tomography imaging as a biomarker for preclinical rheumatoid arthritis: findings of a prospective pilot study. Arthritis Rheum..

[31] Gent YYJ, Ahmadi N, Voskuyl AE, Hoetjes N, Van C, Britsemmer K (2014). Detection of subclinical synovitis with macrophage targeting and positron emission tomography in patients with rheumatoid arthritis without clinical arthritis detection of subclinical synovitis with macrophage targeting and positron emission tomography in. J Rheumatol..

[32] Narayan N, Owen DR, Mandhair H, Smyth E, Carlucci F, Saleem A (2018). Translocator protein as an imaging marker of macrophage and stromal activation in rheumatoid arthritis pannus. J Nucl Med..

[33] Wadsworth H, Jones PA, Chau WF, Durrant C, Fouladi N, Passmore J (2012). [18F]GE-180: a novel fluorine-18 labelled PET tracer for imaging translocator protein 18 kDa (TSPO). Bioorg Med Chem Lett.

[34] Dickens AM, Vainio S, Marjamäki P, Johansson J, Lehtiniemi P, Rokka J (2014). Detection of microglial activation in an acute model of neuroinflammation using PET and radiotracers 11C-(R)-PK11195 and 18F-GE-180. J Nucl Med..

[35] Boutin H, Murray K, Pradillo J, Maroy R, Smigova A, Gerhard A (2015). 18F-GE-180: a novel TSPO radiotracer compared to 11C-R-PK11195 in a preclinical model of stroke. Eur J Nucl Med Mol Imaging..

[36] Airas L, Dickens AM, Elo P, Marjamäki P, Johansson J, Eskola O (2015). In vivo PET imaging demonstrates diminished microglial activation after fingolimod treatment in an animal model of multiple sclerosis. J Nucl Med..

[37] Aletaha D, Neogi T, Silman AJ, Funovits J, Felson DT, Bingham CO (2010). 2010 Rheumatoid arthritis classification criteria: An American College of Rheumatology/European League Against Rheumatism collaborative initiative. Arthritis Rheum..

[38] Prevoo MLL, Van’T Hof MA, Kuper HH, Van Leeuwen MA, Van De Putte LBA, Van Riel PLCM (1995). Modified disease activity scores that include twenty-eight-joint counts development and validation in a prospective longitudinal study of patients with rheumatoid arthritis. Arthritis Rheum..

[39] Owen DR, Yeo AJ, Gunn RN, Song K, Wadsworth G, Lewis A (2012). An 18-kDa translocator protein (TSPO) polymorphism explains differences in binding affinity of the PET Radioligand PBR28. J Cereb Blood Flow Metab..

[40] Cootes TF, Edwards GJ, Taylor CJ (2001). Active appearance models. IEEE Trans Pattern Anal Mach Intell..

[41] Tofts PS (1997). Modeling tracer kinetics in dynamic Gd-DTPA MR imaging. J Magn Reson Imaging..

[42] Tak PP, Smeets TJM, Daha MR, Kluin PM, Meijers KAE, Brand R (1997). Analysis of the synovial cell infiltrate in early rheumatoid synovial tissue in relation to local disease activity. Arthritis Rheum..

[43] Kraan MC, Versendaal H, Jonker M, Bresnihan B, Post WJ, ’t Hart BA (1998). Asymptomatic synovitis precedes clinically manifest arthritis. Arthritis Rheum..

[44] Feeney C, Scott G, Raffel J, Roberts S, Coello C, Jolly A (2016). Kinetic analysis of the translocator protein positron emission tomography ligand [18F]GE-180 in the human brain. Eur J Nucl Med Mol Imaging..

[45] Fan Z, Calsolaro V, Atkinson RA, Femminella GD, Waldman A, Buckley C (2016). Flutriciclamide (18F-GE180) PET: first-in-human PET study of novel third-generation in vivo marker of human translocator protein. J Nucl Med..

[46] Vomacka L, Albert NL, Lindner S, Unterrainer M, Mahler C, Brendel M (2017). TSPO imaging using the novel PET ligand [18F]GE-180: quantification approaches in patients with multiple sclerosis. EJNMMI Res..

[47] Bruijnen STG, Gent YYJ, Voskuyl AE, Hoekstra OS, van der Laken CJ (2014). Present role of positron emission tomography in the diagnosis and monitoring of peripheral inflammatory arthritis: a systematic review. Arthritis Care Res (Hoboken)..

[48] Goerres GW, Forster A, Uebelhart D, Seifert B, Treyer V, Michel B (2006). F-18 FDG whole-body PET for the assessment of disease activity in patients with rheumatoid arthritis. Clin Nucl Med..

[49] Fosse P, Kaiser M-J, Namur G, de Seny D, Malaise MG, Hustinx R (2018). 18F- FDG PET/CT joint assessment of early therapeutic response in rheumatoid arthritis patients treated with rituximab. Eur J Hybrid Imaging..

[50] Chung SJ, Youn H, Jeong EJ, Park CR, Kim MJ, Kang KW (2018). In vivo imaging of activated macrophages by 18F-FEDAC, a TSPO targeting PET ligand, in the use of biologic disease-modifying anti-rheumatic drugs (bDMARDs). Biochem Biophys Res Commun..

